# Family Factors and the Psychological Well-Being of Children and Adolescents with Inflammatory Bowel Disease—An Exploratory Study

**DOI:** 10.3390/children12050575

**Published:** 2025-04-29

**Authors:** Chantal Hieronymi, Kalina Kaul, Jan de Laffolie, Burkhard Brosig, on behalf of Cedata-GPGE AG

**Affiliations:** Department of General Paediatrics and Neonatology, University Children’s Hospital, Justus-Liebig-Universität Gießen, Feulgenstr 12, 35392 Gießen, Germany; chantal.hieronymi@stud.uni-giessen.de (C.H.); kalina.kaul@paediat.med.uni-giessen.de (K.K.); burkhard.brosig@psycho.med.uni-giessen.de (B.B.)

**Keywords:** pediatric inflammatory bowel disease, ulcerative colitis, Crohn’s disease, psychosocial factors, family structure

## Abstract

**Background/Objectives**: The aim of our study was to examine the influence of family structure and the number of siblings on psychological problems and illness-related emotions in children and adolescents with inflammatory bowel disease (IBD) and the relationship between emotional coping in children and parents. **Methods**: CEDNA is a nationwide German online and paper-based questionnaire administered between October 2021 and April 2022. Adolescents with IBD, aged 12 to 17 years, and parents of children aged 0 to 17 years with diagnosed IBD, were included. SAS was used for descriptive statistics and logistic regression analysis was performed using R Studio (PBC; 2023.06.0 + 421). **Results**: 1158 participants (450 adolescents and 708 parents) were included in the study. A two-parent household could not be associated with mental illness as a comorbidity in pediatric IBD patients (*p* = 0.06) but was shown to decrease the risk of sadness (*p* < 0.001), helplessness (*p* < 0.01), feeling left alone and lonely (*p* < 0.05). A single-parent household increased the risk of sadness (*p* = 0.001), helplessness (*p* = 0.002), and loneliness (*p* = 0.006). Having one to two siblings was associated with a lower risk of mental health problems (*p* = 0.03) and reduced anxiety (*p* = 0.005). An association was also found between parents and children’s emotional coping skills. **Conclusions**: Further research on family structure and siblings in pediatric IBD is needed, given the potential impact on children’s psychological well-being.

## 1. Introduction

An increasing incidence of childhood-onset inflammatory bowel disease (IBD) has been reported worldwide [1]. In 2012, the prevalence of pediatric IBD (pIBD) in Germany was 66.29/100.000, making Germany one of the countries with the highest prevalence internationally [1,2]. Common symptoms of pIBD include abdominal pain, diarrhea, rectal bleeding, fatigue, loss of appetite, weight loss, and growth delay [3]. As a chronic and fluctuating disease, pIBD is unpredictable for the sufferer and is associated with potentially embarrassing symptoms (e.g., frequent trips to the bathroom, diarrhea, bloating) that can lead to stigma and shame [4]. Adolescents with IBD are at increased risk for psychosocial impairment, with a higher prevalence of depressive and anxiety symptoms, lower health-related quality of life (HRQoL), and poorer social functioning compared to healthy peers [5,6,7,8]. This can negatively affect adherence to treatment, sleep, perception of abdominal pain [9], and overall disease symptoms [10].

Family members are the main source of support and care for children and adolescents with IBD [11]. Family structure and functioning are possible psychosocial predictors in pIBD [12,13]. Against the background of the increasing number of single parents in Germany, as well as the increasing frequency of separations and new family structures [14], growing up in single-parent or stepfamily households has been associated with a higher risk of emotional and behavioral problems in children and adolescents in Germany [15]. A decrease in self-esteem has been associated with separated parents in adolescent IBD patients [13], but no other studies in pIBD focusing on family structure was found. Greater parental stress, which may occur in single-parent families, was associated with internalising symptoms in pIBD patients. [16]. In a paradigmatic study of type 1 diabetes, adolescents living in single-parent households had a lower quality of life than those living in two-parent households [17]. They also had poorer metabolic control [18,19]. Both could be extrapolated to pediatric IBD patients in terms of reduced treatment adherence, potentially increased disease activity, and reduced quality of life when growing up in a single-parent household. Further, the presence of siblings influences the psychosocial development of children and can affect cognitive and social skills in families [20]. Warm sibling relationships, positive attachment, and older siblings were shown to positively influence social and emotional functioning [21,22]. On the other hand, the recourse dilution theory predicts poorer mental health and intellectual skills as the number of siblings increases [23,24,25]. Parental involvement has been shown to decrease with more siblings, which could affect the care of children with IBD [26]. No relevant literature has been found on the influence of the number of siblings on psychosocial well-being in pIBD patients. Moreover, bidirectional influences of parents’ and children’s coping strategies were found in children with chronic diseases [27]. Emotional dyadic coping reported by parents was associated with a higher quality of life in their children [27]. Also, parents’ illness uncertainty and depressive symptoms were related to internalizing symptoms of adolescent IBD patients [28,29]. Parents and children thus influence each other, which is essential for coping with chronic illness [30].

Based on these findings, this study is the first to examine the influence of family structure and the number of siblings on the prevalence of psychological problems and illness-related emotions in children and adolescents with IBD, as well as the relationship between parents’ and children’s emotional coping strategies.

## 2. Materials and Methods

### 2.1. Participants

Adolescents with diagnosed IBD, aged 12–17 years, and parents of children aged 0–17 years with diagnosed IBD, were included. They participated in the study between October 2021 and April 2022. There were no exclusion criteria for this study.

### 2.2. Procedure

The CEDNA Study is an anonymous survey addressing children and adolescents with IBD and their parents to analyze the informational needs, supply system, communication services, and psychological burden of the disease. The questionnaires were developed by a working group consisting of pediatric gastroenterologists, CED-KQN project scientists, and DCCV e.V. (German Crohn’s and Ulcerative Colitis Association) staff as previously described [31]. The development of the questionnaires took place over a period of more than one year (11/2018–02/2020). The patient questionnaire consisted of 28 questions and the parent questionnaire consisted of 41 questions. The survey results were not formally validated. The paper-and-pencil questionnaires were distributed to the addresses of families who had children diagnosed with IBD and were on the membership list of the DCCV e.V. In addition, questionnaires were sent to the participating patients and their families in the patient registry of the Society for Pediatric Gastroenterology and Nutrition (CEDATA-GPGE) and centers of the GPGE e.V. society to address further outpatient clinics. Paper-based questionnaires were digitalized at the study center in Giessen. In addition, an online questionnaire was created using the LimeSurvey portal. We used self-help groups on social media channels; the websites of DCCV e.V. and GPGE e.V.; the CEDATA, CLARA, and DCCV newsletters; and the websites cedmo.de and abbvie-care. de, and ced-kqn.de to distribute the online questionnaire. The participants did not receive any incentives.

### 2.3. Data Collection

#### 2.3.1. Patient and Parent Characteristics

Sociodemographic data were collected from the patients and their parents. The patient questionnaire included information on age, sex, diagnosis, time since diagnosis, and disease phase. The parental questionnaire included the same variables and additional data on the disease course and parental education. Regarding the diagnosis, we considered Crohn’s disease, ulcerative colitis, and IBD unclassified. Time since diagnosis was categorized as “less than 1 year”, “1 to 2 years”, “3 to 4 years”, “5 to 6 years” and “more than 6 years”. Disease phase was classified as either “flare”, “remission” or “diagnostic phase.” Disease progression was classified into three categories: “sustained remission”, “recurrent flares”, and “increasing activity or active disease”.

#### 2.3.2. Family Structures

The term “family structures” refers to the living environment of the child. We collected information on the parents with whom the child primarily lived, as well as any instances of non-traditional or alternative family arrangements. For a two-parent household, we assumed in the parent questionnaire that the child lived with both biological parents, whereas the patient questionnaire specifically asked about living with both biological parents. To qualify as a single-parent household, the child lived with one biological parent and no partner.

#### 2.3.3. Siblings

Siblings were categorized as present or absent. This category was then subdivided into having one to two siblings and three or more siblings.

#### 2.3.4. Psychological Problems of the Patient

We asked about comorbidities and incorporated data on mental health problems into our database. The presence or absence of psychological problems was assessed. No specific diagnoses were surveyed.

#### 2.3.5. Patient Emotions

Participants’ responses to the question, “Which emotions do you experience when you think about your IBD?” and specific questions about feelings of sadness, anxiety, insecurity, helplessness, exhaustion, loneliness, feeling alone, feeling overwhelmed, nervousness, shame, lack of courage, feeling calm, and believing that “it will be okay” were evaluated.

#### 2.3.6. Patient and Parent Emotional Coping

Parents and children were asked about their ability to cope with their emotions. The responses were: “I am always able”, “I am mostly able”, “I am sometimes able”, and “I am not able to manage my emotions.” The last two responses were categorized as one: “Little to no ability to manage emotions.”

### 2.4. Statistical Analysis

Descriptive statistical analyses were performed using Microsoft Excel (Office 365, version 2022), SAS (version 9.4), and R Studio (PBC; 2023.06.0 + 421). Continuous and categorical variables were defined using descriptive statistics. Continuous variables were evaluated using means, medians, ranges, and standard deviations. Logistic regression analysis was performed using R Studio to determine the influence of family and sibling information on psychological outcomes. Associations between family structure, number of siblings, and disease-related emotions of the children were analyzed. The parent and patient questionnaires were merged and sorted by matching patient IDs to determine the association between parents’ and children’s emotional coping.

Statistical significance was set at *p* < 0.05. Odds ratios were calculated to show a positive or negative association between the variables analyzed. Due to the exploratory nature of the study, statistical significance did not have the same theoretical status as hypothesis-testing designs.

## 3. Results

**Figure 1:**
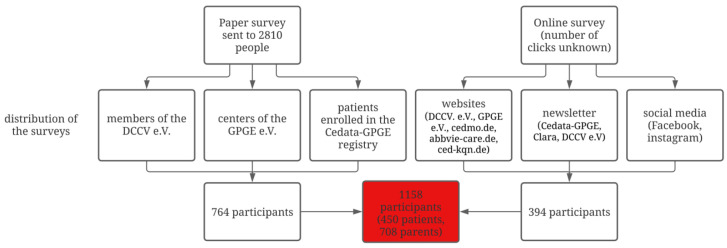
Distribution of the surveys in the CEDNA study (Red: total number of participants).

A total of 1158 participants responded to the paper survey, including 708 parents and 450 patients. Of the 2810 paper surveys sent out, 764 were returned. Therefore, the response rate of the paper questionnaires was 27%. A total of 394 responded using the online questionnaire (see Figure 1). The online response rate could not be determined for data security reasons, and therefore, there are no data on the number of clicks.

### 3.1. Patient and Parent Characteristics

The mean age of the patients who completed the patient questionnaire was 14.8 years (SD 1.5), according to the patient questionnaires. The mean age of the parents was 41–60 years. Among the adolescents, 50.4% were girls, 48.3% were boys, and 1.3% were diverse; 52.4% had Crohn’s disease, 42.2% had ulcerative colitis, and 9.2% had IBD unclassified. The median disease duration was 1–2 years. In total, 75.7% of the patients were in remission, 12.4% had disease activity, and 1.5% were in the diagnostic phase. Most adolescents lived in two-parent households (82.0%), 9.5% lived in single-parent households, and 8.5% lived in other family structures. A total of 83.7% of the adolescents had siblings, and 16.3% had no siblings. No answers or incorrect answers were not included in the descriptive analysis (see Table 1).

### 3.2. Family Structures and Psychological Problems

Looking at the influence of family structures on IBD patients as documented in the patient questionnaire, growing up with both biological parents showed no effect on the prevalence of psychological problems in the child (OR 0.47, CI 0.22–1.07, *p* = 0.06), barely missing significance. Growing up in a single-parent household (OR 1.89, CI 0.76–4.26, *p* = 0.14) or in other non-traditional family structures (OR 1.53, CI 0.43–4.24, *p* = 0.46) did not display a significant effect despite the elevated odds ratios.

Parallel to these findings, growing up in a two-parent household (OR 0.67, CI 0.34–1.48, *p* = 0.29), a single-parent household (OR 1.70, CI 0.74–3.54, *p* = 0.18), and other family structures (OR 0.66, 0.04–3.38, *p* = 0.69) displayed no relevant influences, as seen in the parent questionnaire (see Table 2).

### 3.3. Siblings and Psychological Problems

In the patient questionnaires, neither having siblings (OR 0.60, CI 0.27–1.48, *p* = 0.23) nor having no siblings (OR 1.67, CI 0.68–3.75, *p* = 0.23) had any relevant influence on psychological problems. Having one or two siblings was negatively associated with the prevalence of mental health problems (OR 0.45, CI 0.22–0.95, *p* = 0.03). Having three or more siblings showed no association with mental health problems (OR 2.31, CI 0.81–5.71, *p* = 0.09). The parent questionnaires did not show any relevant influence of the number of siblings on the prevalence of mental health problems in the children (see Table 2).

### 3.4. Family Structures and Emotions

Growing up in a two-parent household was associated with a decrease in the occurrence of certain negative emotions, including sadness (OR 0.36, CI 0.20–0.64, *p* < 0.001), helplessness (OR 0.30, CI 0.13–0.76, *p* = 0.009), loneliness (OR 0.45, CI 0.22–0.94, *p* = 0.029), and feeling left alone (OR 0.37, CI 0.15–1.03, *p* = 0.045) in the children and adolescents. Growing up in a single-parent household had the greatest effect on feelings of helplessness (OR 4.09, CI 1.62–9.91, *p* = 0.002), sadness (OR 2.69, CI 1.46–4.92, *p* = 0.001), and loneliness (OR 2.76, CI 1.30–5.62, *p* = 0.006). Just missing significance was the effect of the single-parent household on feelings of anxiety (OR 1.96, CI 0.96–3.83, *p* = 0.055), insecurity (OR 1.70, CI 0.94–3.05, *p* = 0.076), and feeling left alone (OR 2.56, CI 0.87–6.74, *p* = 0.067) in the IBD patients. For alternative family structures, a lower *p*-value was found only for feeling left alone, not reaching statistical significance (OR 2.90, CI 0.87–6.74, *p* = 0.073). No associations were found between family structure and feelings of exhaustion, feeling overwhelmed, nervousness, shame, lack of courage, or the feeling that “it will be fine” (see Table 3).

### 3.5. Siblings and Emotions

Having siblings had a reducing effect on feelings of anxiety about the illness (OR 0.45, CI 0.23–0.88, *p* = 0.016). When examining the number of siblings, having one to two siblings showed a significant reduction in anxiety (OR 0.43, CI 0.24–0.79, *p* = 0.005) and a positive association with the feeling that “it will be fine” (OR 2.19, CI 1.14–4.13, *p* = 0.016). The effect of having one or two siblings on feelings of insecurity did not reach statistical significance (OR 0.65, CI 0.40–1.06, *p* = 0.083). Having three or more siblings had a negative impact on the feeling that “it will be okay” (OR 0.28, CI 0.13–0.66, *p* = 0.002). Having no siblings was positively associated with anxiety (OR 2.24, CI 1.14–4.29, *p* = 0.016). No association was found between siblings and feelings of sadness, helplessness, exhaustion, feeling left alone, loneliness, feeling calm, nervousness, shame, or lack of courage (see Table 3).

### 3.6. Parent–Child Emotional Coping

Parents with little or no ability to manage their emotions showed a significant influence on how their children could process their emotionality (OR 7.8, CI 1.92–27.20, *p* = 0.002). Low or no parental ability to manage emotions was negatively related to children who were always able to manage their emotions (OR 0.3, CI 0.09–0.85, *p* = 0.03). The parents’ ability to mostly manage their emotions had a positive effect on the children’s ability to manage their emotions most of the time (OR 1.9, CI 1.13–3.21, *p* = 0.02) and a negative effect on the children’s ability to manage their emotions all of the time (OR 0.52, CI 0.31–0.85, *p* = 0.01). Parents who were always able to cope with their emotions showed a positive association with children being able to cope (OR 2.6, CI 1.56–4.40, *p* < 0.001), a negative association with children mostly able to cope (OR 0.49, CI 0.28–0.82, *p* = 0.008), and a negative association with children who had little or no ability to cope (OR 0.25, CI 0.06–0.82, *p* = 0.037) (see Table 4).

## 4. Discussion

Illness-related psychological symptoms were found to be associated with family structure and the number of siblings. This study found no significant association between family structure and psychological comorbidity in pediatric IBD patients but found a significant effect of the number of siblings on psychological problems in affected children. In the analysis of emotional coping skills of parents and children, a significant correlation was demonstrated.

### 4.1. Family Structures and Psychological Problems/Disease-Related Emotions

Among the children and adolescents with IBD in our study, living in a family with both biological parents was not associated with fewer mental health problems. For youth living with one parent or in alternative family structures, no associations with psychological problems could be made. Only one study focusing on family structure and a psychological measure in pIBD patients demonstrated lower self-esteem in children with separated parents [13]. Self-esteem has been reported to predict HRQoL, emotional functioning, and anxiety in pediatric IBD patients [32], making low self-esteem a possible precursor of mental illness. More literature was found on family structure and another chronic illness, namely type 1 diabetes. Living with both biological parents was related to a higher HRQoL in children with type 1 diabetes, and living with separated parents was related to a lower HRQoL [17]. This study used a different measure, not assessing the diagnosis of a mental illness, but measuring emotional, social, and physical health directly related to the disease. Consequently, the outcome of our study cannot be directly compared with that of the HRQoL study in pediatric patients with diabetes. It is conceivable that if we measured HRQoL in children and adolescents with IBD, an association between family structure and HRQoL could be made. There is a possible bias in our study due to the difference in the number of single-parent (9.6%) to two-parent households (82%), which could make the results more difficult to interpret.

In addition, we assessed children’s emotions related to the disease. Significantly lower rates of sadness, loneliness, helplessness, and the feeling of being left alone were demonstrated in children growing up with both biological parents. An increase in sadness, helplessness, and loneliness was found among children living in single-parent households. Increased internalizing of symptoms in children with IBD have been associated with higher parental distress [16], which may work bidirectionally, with increased parental stress negatively influencing the internalizing of symptoms in patients. No literature was found on stress in single parents raising a child with IBD. The feeling of helplessness in children could be related to single parents having to work and simultaneously take care of a child with IBD, which can be overwhelming and can lead to feelings of helplessness in parents. As bidirectional influences of parents’ and children’s psychological states have been proven [27], this could contribute to the increase of helplessness in children.

### 4.2. Siblings and Psychological Problems/Disease-Related Emotions

Having one to two siblings is associated with a significantly lower risk of mental health problems in children with IBD. The presence of siblings, specifically having one or two siblings, was associated with less disease-related anxiety and a more positive future outlook. Having three or more siblings was negatively associated with a positive future outlook, while having no siblings was associated with increased anxiety.

The decreasing effect of having one or two siblings on psychological problems in pIBD patients may be a consequence of warm sibling relationships and the early development of social skills with positive interactions [22]. As attachment behavior in relationships can regulate emotional functioning [33], it is possible that positive sibling attachment helps to build healthy emotion-management strategies. Warm sibling relationships and less sibling conflict have also shown to positively affect children’s internalizing and externalizing of symptoms [22], being a possible reason for the reduction in disease-related anxiety.

The significant association of having three or more siblings with a less positive future outlook could be explained by the results of a Turkish study of fourth-graders. Children with two to four siblings had a higher perception of parental acceptance and involvement than those with five or more siblings [26]. Difficulties may arise in the parent’s management of not only caring for each child individually and building a warm relationship but also caring for a child with a chronic illness. The expected parental care of children with IBD and the parent–child relationship may not meet the child’s needs due to the large number of siblings, which has been previously described with the recourse dilution model [23,25].

### 4.3. Emotional Coping Abilities of Parents and Children

By merging the patients’ and parents’ survey data, we were able to link the emotional coping abilities of the children to their parents. Parents and children influencing each other has been demonstrated across studies on pediatric IBD, and therefore support our results, making this important to consider in the future management of pIBD patients [27,28,29].

### 4.4. Limitations and Strenghts

The first limitation is the cross-sectional design, which impedes statements about the directions of influences and changes over time. Second, our study is exploratory and lacks a hypothesis-based analysis; therefore, significance is not assured. Third, the analog response rate is low. This makes response bias possible, and, to increase responses, no extensive workup with longer questionnaires or other measures was performed due to data security and the anonymity of the study. Fourth, due to the survey development process, we did not use extensively validated measures. This makes it difficult to compare the results of our study with the existing literature, and the lack of validation reduces the power of the findings; however, few validated tools are available for this specific topic. The length of this voluntary survey needed to be limited, and further development is needed. Fifth, a possible discrepancy between the definition of the two-parents household in the patient and parent questionnaire exists due to differing response options, as stated in the methods part, Section 2.

A strength of our study is the large multicentered sample size. The parameters of the patients suggested a representative cohort of patients with pIBD in Germany. Another strength is the questioning of both the patients and their parents. Comparisons could be made between patient and parent reports, and bidirectional influences could be demonstrated, both of which are underrepresented in the literature and will guide further studies in our group.

## 5. Conclusions

This study is the first to explore the impact of family structure and number of siblings on psychological outcomes in children with IBD and the relationship between parents’ and children’s emotional coping. Family structure has been shown to influence disease-related emotions (e.g., sadness, helplessness, loneliness), with a two-parent household decreasing the risk of these emotions and a single-parent household increasing the risk. However, family structure was not associated with the presence of reported mental health problems in the children. Another facet of family structure, having siblings, showed a decrease in anxiety; having one to two siblings was associated with a lower risk of mental health problems and anxiety and a more positive future outlook, whereas having three or more siblings was negatively associated with a positive future outlook. Furthermore, a relationship between parents’ and children’s emotional coping abilities was demonstrated. To gain a clearer understanding of these family influences, further research, including the development of validated, structured measures, patient-reported outcomes, and a longitudinal design, would be needed, e.g., inside the patient registry CEDATA GPGE.

## Figures and Tables

**Table 1:** Descriptive analysis of the demographic data and disease variables of patients and parents.

Child/Adolescent Data	Patient Survey	Parent Survey
*Child’s age (yr)*	*n* = 365	*n* = 614
Mean (SD)	14.8 (1.5)	12.9 (3.3)
*Gender (%)*	*n* = 381	*n* = 615
Female	50.4	48.0
Male	48.3	51.9
Diverse	1.3	0.2
*Family structure (%)*	*n* = 377	*n* = 573
Two-parent household	82.0	86.2
Single-parent household	9.5	11.2
Other	8.5	2.6
*Siblings (%)*	*n* = 381	*n* = 570
No siblings	16.3	19.5
Has siblings	83.7	80.5
1–2 siblings	74.5	71.2
≥3 siblings	9.2	9.3
*Diagnosis (%)*	*n* = 410	*n* = 613
Crohn’s disease,	52.4	51.2
Ulcerative colitis	42.2	40.8
IBD unclassified	5.4	8.0
*Disease duration (yr)*	*n* = 410	*n* = 616
Median	1–2	1–2
*Disease phase (%)*	*n* = 403	*n* = 611
Remission	75.7	68.4
Flare up	12.4	15.6
Diagnosis	1.5	4.3
I don’t know	10.4	11.8
*Disease course of the child (%)*		*n* = 603
Sustained remission		54.4
Recurrent flares		25.5
Active disease		17.4
Increasing activity		2.7
**Parent data**		
*Parental age group (yr)*		*n* = 574
Median		41–60
*Educational attainment of the parents (%)*		*n* = 560
University degree		25.4
Apprenticeship or vocational training		51.8
Polytechnic degree		17.9
None		5.0

SD: standard deviation; Yr: year, based on [31].

**Table 2:** Impact of family structures and siblings on the presence of psychological problems in the children and adolescents.

	Patient Survey		Parent Survey	
	OR (CI)	*p*-Value	OR (CI)	*p*-Value
Two-parent household	0.47 (0.22–1.07)	0.060 *	0.67 (0.34–1.48)	0.294
Single-parent household	1.89 (0.76–4.26)	0.144	1.70 (0.74–3.54)	0.177
Other family structure	1.53 (0.43–4.24)	0.457	0.66 (0.04–3.38)	0.690
Has siblings	0.60 (0.27–1.48)	0.233	0.90 (0.46–1.90)	0.772
1–2 siblings	0.45 (0.22–0.95)	0.032 *	0.70 (0.39–1.30)	0.249
≥3 siblings	2.31 (0.81–5.71)	0.088 *	1.77 (0.73–3.80)	0.170
No siblings	1.67 (0.68–3.75)	0.233	1.11 (0.53–2.16)	0.772

OR: odds ratio, CI: confidence interval, * *p* < 0.1.

**Table 3:** Influence of family structures and siblings on the illness-related emotions of the adolescents—patient survey.

	Sad	Afraid	Insecure	Helpless	Exhaust-ed	Left Alone	Over-Whelmed	Nervous	Shame	Loneli-ness	Loss of Courage	Calm	Fine
Two-parent household	0.36 ***	0.58	0.69	0.30 **	0.63	0.37 *	0.70	0.89	0.78	0.45 *	0.68	1.11	1.26
One-parent household	2.69 **	1.96 *	1.70 *	4.09 **	1.65	2.56 *	1.38	0.95	1.61	2.76 **	1.81	0.80	0.64
Other family stuctures	1.48	0.99	0.55	0.48	0.76	2.90 *	0.75	0.41	0.70	1.11	1.12	2.06	2.23
Has siblings	0.66	0.45 *	0.68	0.92	0.92	1.17	0.73	1.60	1.80	1.27	2.01	1.16	1.19
1–2 siblings	0.77	0.43 **	0.65 *	0.61	0.78	0.83	0.67	0.99	1.36	0.90	0.89	1.42	2.19 *
≥3 siblings	0.91	1.66	1.42	2.34	1.53	1.80	1.47	1.71	1.15	1.72	2.48	0.58	0.28 **
No siblings	1.51	2.24 *	1.47	1.09	1.09	0.85	1.37	0.62	0.56	0.78	0.50	0.86	0.84

Given in odds ratio, * *p* < 0.1, ** *p* < 0.01, *** *p* < 0.001; Fine: “I think it will be fine”.

**Table 4:** Coping with emotions—association of parents’ emotional coping ability with their child’s emotional coping ability.

	Parents					
Patients	Little to None		Mostly		Always	
	OR(CI)	*p*-Value	OR(CI)	*p*-Value	OR(CI)	*p*-Value
Little to none	7.77 (1.92–27.20)	0.002 **	1.35 (0.47–3.95)	0.578	0.25 (0.06–0.82)	0.037 *
Mostly	1.38 (0.45–3.97)	0.551	1.89 (1.13–3.21)	0.017 *	0.49 (0.28–0.82)	0.008 **
Always	0.29 (0.09–0.85)	0.030 *	0.51 (0.31–0.85)	0.010 *	2.60 (1.56–4.40)	<0.001 ***

OR: odds ratio, CI: confidence interval, * *p* < 0.1, ** *p* < 0.01, *** *p* < 0.001.

## Data Availability

The data underlying this article, including anonymized patient data, will be shared upon reasonable request with the corresponding author.

## Abbreviations

The following abbreviations are used in this manuscript:

CI	Confidence interval
DCCV e.V.	Deutsche Morbus Crohn/Colitis Ulcerosa Vereinigung
GPGE e.V	Gesellschaft für Pädiatrische Gastroenterologie und Ernährung
HRQoL	Health-related quality of life
IBD	Inflammatory bowel disease
pIBD	Pediatric inflammatory bowel disease
OR	Odds ratio
SD	Standard deviation
yr	Year
